# Patient perspective on task shifting from general practitioners to medical practice assistants – a quantitative survey in Germany

**DOI:** 10.1186/s12875-023-02211-5

**Published:** 2023-11-25

**Authors:** Doreen Kuschick, Marius Tibor Dierks, Ulrike Grittner, Christoph Heintze, Lisa Kümpel, Burgi Riens, Liliana Rost, Konrad Schmidt, Daniel Schulze, Kahina Toutaoui, Florian Wolf, Susanne Döpfmer

**Affiliations:** 1 Charité - Universitätsmedizin Berlin, corporate member of Freie Universität Berlin and Humboldt-Universität zu Berlin, Institute of General Practice and Family Medicine, Charitéplatz 1, Berlin, 10117 Germany; 2 Universitätsklinikum Jena, Friedrich-Schiller-Universität Jena, Institute of General Practice and Family Medicine, Bachstraße 18, Jena, 07743 Germany; 3 Charité – Universitätsmedizin Berlin, corporate member of Freie Universität Berlin and Humboldt-Universität zu Berlin, Institute of Biometry and Clinical Epidemiology, Charitéplatz 1, Berlin, 10117 Germany

**Keywords:** Delegation, General practice, General practitioner, Medical practice assistants, Patients’ perspective, Questionnaire

## Abstract

**Introduction:**

Various developments result in increasing workloads in general practices. New models of care and a restructuring of the division of tasks could provide relief. One approach is to extend the delegation of medical tasks from general practitioners (GPs) to medical practice assistants (MPAs). So far, there has been a lack of information about specific situations in which patients are willing to be treated exclusively by MPAs.

**Methods:**

In three German federal states, patients who visited a general practice were surveyed exploratively and cross-sectionally with a self-designed, paper-based questionnaire. The data were analysed descriptively and multivariate. A mixed binary logistic regression model was calculated to account for cluster effects at practice level (random intercept model). The dependent variable was patients' acceptance of task delegation.

**Results:**

A total of 1861 questionnaires from 61 general practices were included in the analysis. Regarding the current problem/request, a total of 30% of respondents could imagine being treated only by MPAs. Regarding theoretical reasons for consultation, more than half of the patients agreed to be treated by MPAs. According to the regression model, MPAs were preferred when patients were younger (10-year OR = 0.84, 95%-CI [0.75, 0.93]) or had a less complicated issue (OR = 0.44, 95%-CI [0.26, 0.8]). For four current problems/requests (“acute complaints” OR = 0.27, 95%-CI [0.17, 0.45], “routine health check” OR = 0.48, 95%-CI [0.3, 0.79], “new problem” OR = 0.13, 95%-CI [0.06, 0.28], “known problem” OR = 0.16, 95%-CI [0.1, 0.27]) patients prefer to be treated by GPs instead of MPAs.

**Discussion:**

For the first time, statements could be made on patients’ acceptance of task delegation in relation to current and theoretical reasons for treatment in general practices in Germany. The discrepancy in response behaviour on a theoretical and individual level could be explained by different contexts of questions and differences at practice level. Overall, patients seem to be open to increased delegation of medical tasks, depending on the reason for treatment. Selection and response biases should be considered in the interpretation.

**Conclusion:**

The results are not completely opposed to an extension of task delegation. Further interventional studies could provide information on the possible effects of expansion of delegable tasks.

## Introduction

The ageing of society in many countries all over the world [1, 2], the associated increase of chronic diseases and multimorbidity [3, 4], the resulting rise in the complexity of care and particularly in Germany the increasing shortage of qualified staff in healthcare [5] are placing an increased workload on general practices. A possible solution for workload reduction is the assignment of physicians’ activities to medical practice assistants (MPAs) [6, 7]. MPAs are the predominant assistant profession in German practices. Their qualification is based on a three-year vocational training program. They work under supervision, and the full responsibility remains with the general practitioners (GPs). According to the “Agreement on the delegation of medical services to non-medical staff in ambulatory healthcare” defined medical tasks can be delegated to MPAs [8]. In contrast, the assignment of medical tasks to assisting professions including transfer of responsibility is not allowed in German practices [8, 9]. Experience with the transfer of responsibility to other non-physician health care personnel is practiced mostly within a few pilot projects [10]. A more active role of non-physician practice staff beyond supporting administrative work and delivering routine preventive services is not common in Germany yet [11]. In an interview study on the acceptance of delegation among GPs and MPAs it was found that MPAs were open-minded about medical tasks being delegated to them whereas GPs were sceptical or even negative [12].

In the United Kingdom, the United States, and the Netherlands for example, so-called “Nurse Practitioners (NPs)” or “Physician Associates/Assistants (PAs)” have already been independently performing activities of GPs for several years [13–15]. Redaélli et al. conclude in their international literature review that non-medical staff have a high potential to relieve the burden on outpatient care [16]. They highlight the significant patient satisfaction with the services as well as the safety of the services provided by non-physician medical staff. Even newer international studies conclude that NPs/PAs generally contribute to the continuity of care [15, 17–19]. Accessibility to patients is shown to be better [20], NPs/PAs spend more time with patients [21–23] and carry out more examinations than GPs [24, 25]. The reasons for GPs to employ NPs/PAs range from reducing their own workload to improving the quality of care to the opportunity to expand their patient base and/or increase the range of services they offer [15, 26].

Regarding patient perspective, the literature indicates a general acceptance and satisfaction with extended non-physician care [14, 20, 23, 24, 27, 28]. In particular, the takeover of home visits by trained MPAs is highly appreciated [29], although in international comparison, some groups of patients (e.g., people with chronic diseases) prefer medical care by GPs during home visits [22]. The European survey by Ruggeri et al. showed that patients with breast cancer are very satisfied, while patients with type 2 diabetes or heart disease are less satisfied with the care provided by new professional groups [30]. In contrast, a regional survey of chronically ill patients aged 65 and older in Baden-Württemberg (a federal state in Germany) revealed a high level of acceptance and trust in care provided by MPAs [31]. In 2017, the annual nationwide telephone survey of insured patients conducted by the National Association of Statutory Health Insurance Physicians (KBV) in Germany revealed that for minor or chronic illnesses, care by trained MPAs would be conceivable for more than half of the respondents [32]. Patients and also caregivers of dementia patients stated as well that they would benefit from trained MPAs taking over medical activities [33].

Whether the delegation of medical tasks should be further expanded has been controversially discussed for many years in Germany [34]. Previous studies showed a heterogeneous picture from the perspective of GPs and MPAs [12, 26, 34–37]. This was further investigated in the context of the present study in German GP practices [38]. The aim of this study is to explore patients’ perspective on a possible expansion of delegable services and tasks. Since – according to literature – consultation issues seem to impact patients´ perspectives, we addressed the following research question: For which consultation purposes can patients in general practices imagine being treated exclusively by MPAs without the involvement of GPs?

## Method

### Design, participants, and recruitment

**Fig. 1:**
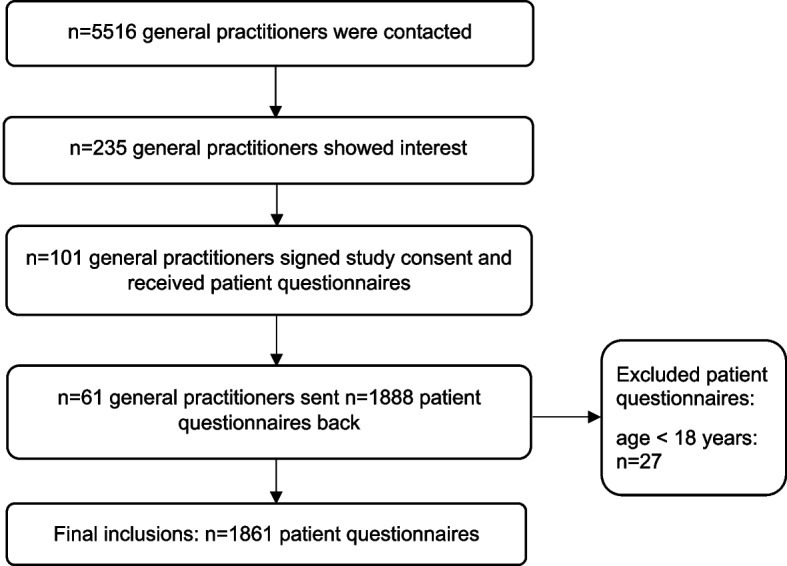
Flowchart study participation and sample

As part of the practice-based research network “RESPoNsE” (RESearch Practice Network East: https://forschungspraxennetz.charite.de/) funded by the German Federal Ministry of Education and Research (BMBF), a project on attitudes towards the assignment of medical tasks to MPAs from the perspective of GPs, MPAs and patients was carried out. The planning of the project and the development of the questionnaires was conducted with the involvement of the practice advisory board of the RESPoNsE network. The results of the questionnaire survey conducted in summer 2021 among all Statutory Health Insurance-accredited GPs in Berlin, Brandenburg, and Thuringia (*n* = 5516, Fig. 1) and their employed MPAs are reported elsewhere [38]. Participating practices were asked to also take part in the subsequent explorative and anonymous cross-sectional survey among patients of GP practices. Due to the explorative nature of the study, no sample size calculation or power calculation was performed. The feasible number of questionnaires to be completed within the given time of two week was discussed with our practice advisory board. Fifty questionnaires were sent to each practice accordingly. In order to participate in the study it was not requested to provide a minimum number of completed questionnaires.

The self-designed, double-sided printed German-language questionnaire was developed based on literature research of comparable studies and on the GP/MPA questionnaire [38]. The piloting of the questionnaire was carried out in two steps: First GPs (*n* = 7) and non-physician colleagues (*n* = 7) as well as two medical layperson were asked to test the questionnaire. As a result, changes were made to the questionnaire. Secondly, the survey procedure (handing out, assigning patients, answering, and returning of the questionnaires) as well as the handling and comprehensibility of all study documents were piloted in one general practice. At this, questionnaire items were piloted again, this time by patients of the practice. No further changes were necessary.

Data collection among participating practices was performed during a two-week survey period in September 2021. The practice teams were informed that they should distribute the questionnaires randomly to the patients during the survey period and not favour certain subgroups of patients or individuals.

The key patient inclusion criteria were sufficient knowledge of the German language and a minimum age of 18 years.

### Measurement

The questionnaire consisted of 18 questions. In addition to the collection of sociodemographic patient data (six questions: age, gender, federal state, region of residence, employment status, educational level), information was collected on the practice visit (four questions: visit with appointment or not, current reason for consultation, urgency and complexity of the issue) and on patients’ state of health (three questions: presence of a chronic disease, state of health in the last two weeks and compared to six months ago). Patients were also asked how long they had been receiving treatment at the practice, how often they had visited the practice in the last twelve months, and to what extent the Corona pandemic had had an influence on the frequency of their practice visits (three questions). Regarding the attitude towards delegation of medical tasks (acceptance of task delegation), the patients were able to mark whether it was absolutely necessary for them to consult their GP for their current problem/request or whether they could also be cared exclusively by MPAs (one question). This assignment could only be made once regarding the current problem/request, even if the patients had more than one problem. Furthermore, the willingness of the patients to be treated by MPAs was asked based on five predefined theoretical reasons for treatment (1. Repeat/follow-up prescription, 2. Sick leave due to a slight cold, 3. Referral, 4. Routine health check, 5. Uncomplicated problem, e.g. a slight cold or vaccination against influenza), each with three possible answers (yes/maybe/no). In general we avoided using the term “delegation” as it is probably not familiar to many patients. Instead, we described the process with easier words (“For my present concern, do I absolutely have to speak to the GP, or could the MPAs help without me talking to the GP?”). The questionnaires were pseudonymised at practice level to provide each practice with the results of their patients compared to the total sample of the study. This possibility of comparison was intended to be an additional incentive for participating practices.

### Data analysis

The analyses conducted were exploratory. Only valid responses were included in the analyses, and no imputation of missing values was performed. Patient characteristics, subjective health status, and practice visit data were analysed descriptively using IBM SPSS Statistics software (version 28.0). To quantify associations between the dependent variable (acceptance of task delegation) and relevant patient characteristics, characteristics of practice visit, and the current problem/request, a mixed binary logistic model (random intercept model) was calculated with the statistical program R version 4.2.1, package Ime4 (1.1–30). In the mixed model, practice ID was included as a random effect (random intercept model). This model was used, as differences between the practices had to be acknowledged (cluster effects at the practice level: e.g., staff and/or structural differences between the practices). The influence of each practice on patient responses can be determined using intraclass correlation (ICC). The dependent variable (acceptance of task delegation) was assessed by the following question: “For my present concern, do I absolutely have to speak to the GP, or could the MPAs help without me talking to the GP?” (coded as GP = 0; MPA = 1). In preparation for analysis, some independent variables were dichotomised or combined. Odds ratios (ORs) and 95% confidence intervals (CIs) were calculated. A two-sided significance level of α = 0.05 was used. No adjustment for multiple testing was made in this exploratory analysis. Thus, *p*-values should be interpreted with caution. The interpretation of the results was based on ORs and 95%-CIs.

### Ethical approval

Positive ethics votes were available from all three federal states before the start of the study (Berlin: EA1/025/21, Brandenburg: AS34(bB)/2021, Thuringia: 2021–2176-Bef).

## Results

### Sample and general information

Of the contacted GPs (*n* = 5516) 235 showed interest (response rate 4.3%, Fig. 1) in the patient survey. A total of 101 GPs (Thuringia *n* = 19, Berlin *n* = 44, Brandenburg *n* = 38) signed the consent form for study participation (Fig. 1). A total of 5.050 questionnaires (50 per practice) were sent to these practices. A total of 61 GPs with 1861 valid patient questionnaires participated (Fig. 1). Referring to all GPs who had the opportunity to participate and who were contacted, 1.1% participated. On average, participating practices returned 31 questionnaires (SD 17, min/max 2/50). Of the questionnaires received (*n* = 1888), 27 were excluded due to the age of the participants (younger than 18 years) (Fig. 1).

More than half of the study participants were female (58.0%) with an average age of 55 years (Table 1). Overall, 50.3% rated their health status as good or very good (Table 1). The detailed characteristics of the study participants are shown in Table 1. At the time of the survey, 61.0% of the patients came to the practice with an appointment (Table 2). More than half of the respondents considered their problem/request to be very or rather urgent (58.3%), 23.8% of the respondents considered their problem/request to be very or rather complicated (Table 2). The most frequently cited reasons for consulting the practice were (multiple responses were possible): acute complaints (34.1%), known problem (28.0%), prescription (28.2%), and routine health check (24.5%) (Table 2). The most frequently mentioned free text answers to the question about other reasons could be categorised as “routine health check” (e.g., blood sampling, control/preventive examinations) and “existing or new problem” (e.g., evaluation of findings, health complaints).

**Table 1:** Participant characteristics

Variables	Thuringia *N* = 532 n/%	Berlin *N* = 648 n/%	Brandenburg *N* = 681 n/%	Total *N* = 1861 n/%
**Age,** Mean (SD)	55.7 (17.6)	54.5 (18.8)	54.6 (17.9)	54.9 (18.1)
Missings	23	72	69	164
**Gender**				
Male	223/42.4	259/41.8	267/41.3	749/41.8
Female	302/57.4	359/57.9	379/58.6	1040/58.0
Diverse	1/0.2	2/0.3	1/0.2	4/0.2
Missings	6	28	34	68
**Residence (federal state in Germany)**				
Berlin	0.0	572/96.3	16/2.6	588/33.9
Brandenburg	1/0.2	19/3.2	601/97.1	621/35.8
Thuringia	506/96.6	1/0.2	1/0.2	508/29.2
Other	17/3.2	2/0.3	1/0.2	20/1.2
Missings	8	54	62	124
**Residential region**				
Metropolis (> 100000 inhabitants)	78/15.2	554/95.4	95/14.8	727/41.9
City (20000 up to 100000 inhabitants)	63/12.3	9/1.5	173/27.0	245/14.1
Town (5000 up to 20000 inhabitants)	110/21.5	6/1.0	175/27.3	291/16.8
Village (< 5000 inhabitants)	261/51.0	12/2.1	198/30.9	471/27.2
Missings	20	67	40	127
**Employment status**				
Pupil	1/0.2	5/0.8	4/0.6	10/0.6
Professional training	14/2.7	10/1.6	16/2.5	40/2.2
Student	11/2.1	18/2.9	11/1.7	40/2.2
Employed	276/53.7	284/45.3	329/50.6	889/49.6
Retired	194/37.7	222/35.4	252/38.8	668/37.3
Job seeking/not employed	10/1.9	68/10.8	23/3.5	101/5.6
Other	8/1.6	20/3.2	15/2.3	43/2.4
Missings	18	21	31	70
**Highest level of education**				
No degree (yet)	5/1.0	19/3.1	9/1.4	33/1.9
Elementary school	63/12.5	115/18.5	74/11.7	252/14.4
Middle school	133/26.5	152/24.5	104/16.5	389/22.2
General qualification for university entrance	48/9.6	76/12.3	60/9.5	184/10.5
Professional training	130/25.9	106/17.1	203/32.2	439/25.0
University Degree	123/24.5	152/24.5	181/28.7	456/26.0
Missings	30	28	50	108
**Duration as patient in general practice**				
< 1 year	54/10.5	72/11.5	65/9.9	191/10.6
1-4 years	125/24.4	160/25.5	174/26.5	459/25.5
5-9 years	75/14.6	130/20.7	141/21.5	346/19.3
10 years or more	259/50.5	265/42.3	277/42.2	801/44.6
Missings	19	21	24	64
**At least one chronic condition**				
Yes	340/67.6	382/65.0	369/59.7	1091/63.8
No	138/27.4	172/29.3	203/32.8	513/30.0
Do not know	25/5.0	34/5.8	46/7.4	105/6.1
Missings	29	60	63	152
**Health condition in the last two weeks**				
Very good	56/10.8	65/10.5	75/11.6	196/11.0
Good	213/41.2	236/38.2	251/39.0	700/39.3
Medium	177/34.2	230/37.2	233/36.2	640/36.0
Bad	66/12.8	73/11.8	69/10.7	208/11.7
Very bad	5/1.0	14/2.3	16/2.5	35/2.0
Missings	15	30	37	82
**Current health condition compared with six months ago**				
Much better	28/5.5	32/6.2	15/2.7	75/4.8
Better	68/13.4	99/19.2	66/12.1	233/14.9
Unchanged	309/61.1	260/50.4	330/60.4	899/57.3
Worse	87/17.2	107/20.7	116/21.2	310/19.8
Much worse	14/2.8	18/3.5	19/3.5	51/3.3
Missings	26	132	135	293
**Frequency of visits to GP in the last twelve months**				
0–1	97/18.9	112/17.9	134/20.4	343/19.1
2–4	278/54.3	336/53.7	378/57.6	992/55.3
5–12	114/22.3	152/24.3	121/18.4	387/21.6
> 12	23/4.5	26/4.2	23/3.5	72/4.0
Missings	20	22	25	67
**Impact of the Corona pandemic on frequency of practice visits in the last twelve months**				
I was in the practice more often	35/6.8	38/6.3	39/6.1	112/6.4
I was in the practice less frequently	65/12.6	107/17.6	85/13.4	257/14.6
No change	396/76.7	440/72.4	484/76.2	1320/75.0
Do not know	20/3.9	23/3.8	27/4.3	70/4.0
Missings	16	40	46	102

**Table 2:** Details of the current practice visit

Variables	Thuringia *N* = 532 n/%	Berlin *N* = 648 n/%	Brandenburg *N* = 681 n/%	Total *N* = 1861 n/%
Practice visit without appointment	210/40.8	247/40.9	234/35.9	691/39.0
Practice visit with appointment	305/59.2	357/59.1	417/64.1	1079/61.0
Missings	17	44	30	91
**Urgency of current problem/request**				
Very urgent	114/22.1	117/20.5	84/13.9	315/18.6
Rather urgent	210/40.7	207/36.2	254/42.1	671/39.7
Less urgent	148/28.7	171/29.9	192/31.8	511/30.2
Not urgent	44/8.5	77/13.5	73/12.1	194/11.5
Missings	16	76	78	170
**Complexity of current problem/request**				
Very complicated	22/4.4	20/3.8	21/3.8	63/4.0
Rather complicated	96/19.0	107/20.2	111/20.1	314/19.8
Less complicated	221/43.8	202/38.0	196/35.6	619/39.0
Not complicated	165/32.7	202/38.0	223/40.5	590/37.2
Missings	28	117	130	275
**Current problem/request**				
Acute complaints	183/34.7	189/33.9	215/33.8	587/34.1
Routine health check	109/20.7	149/298.6	155/24.2	413/24.5
New problem	70/13.3	68/13.0	83/13.3	221/13.2
Known problem	152/28.8	167/30.9	159/24.9	478/28.0
Vaccination/vaccination advice	73/13.8	107/20.2	149/23.8	329/19.5
Prescription	174/33.0	171/31.6	134/21.3	479/28.2
Sick leave	91/17.2	86/16.2	93/14.7	270/16.0
Referral	65/12.3	33/6.4	48/7.7	146/8.8
Other medical certificate	28/5.3	21/4.1	25/4.0	74/4.4
Other reasons	43/8.1	79/14.9	53/8.5	175/10.4
**For my present concern…**				
…do I absolutely have to speak to the GP	367/74.7	397/67.7	435/69.6	1199/70.4
…MPAs could help me without talking to the GP	124/25.3	189/32.3	190/30.4	503/29.6
Missings	41	62	56	159

### Delegation acceptance in relation to the current practice visit

**Fig. 2:**
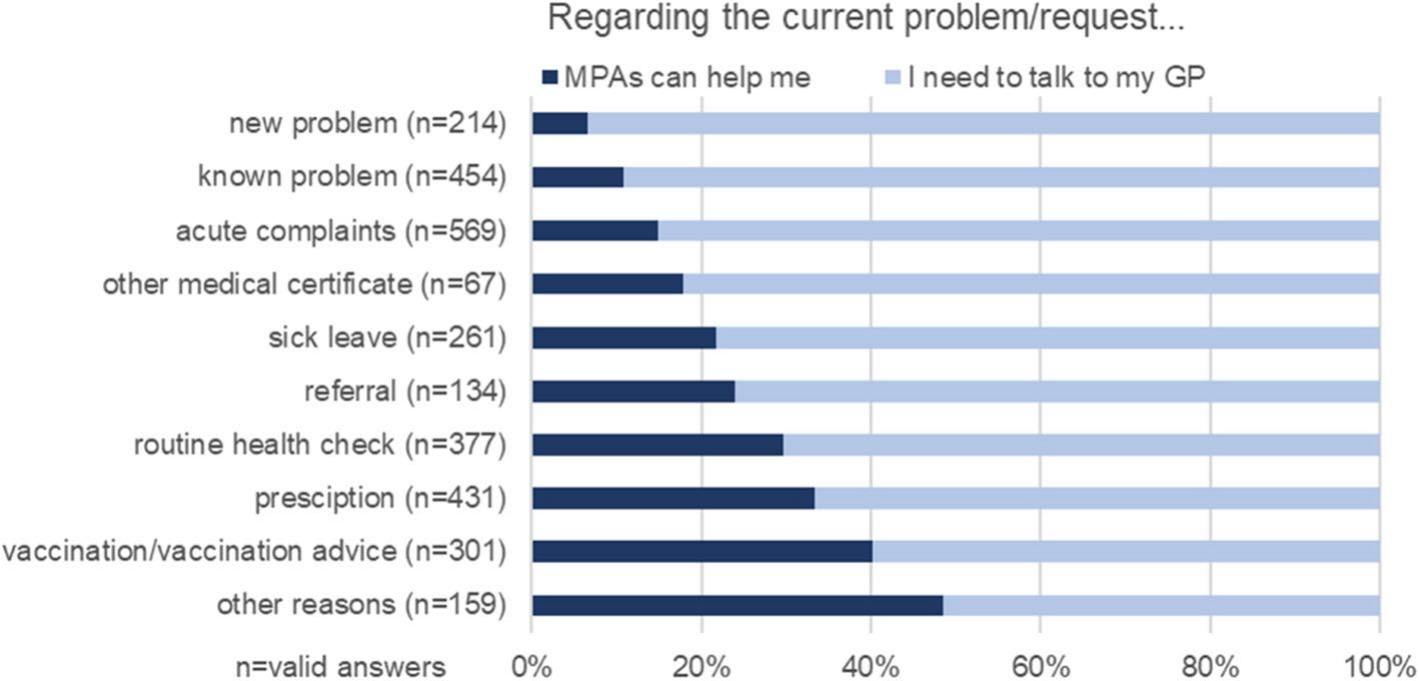
Patients’ task delegation acceptance in relation to their current problem/request

Patients’ acceptance of speaking exclusively with MPAs and not consulting GPs regarding their current problem/request varied at the level of the federal states between 25.3% in Thuringia and 32.3% in Berlin (Table 2). For the entire data set, this applied to 29.6% of respondents (Table 2). When asked about the current reason for visiting the practice, multiple answers were possible. Patients mainly wanted to see a GP for acute complaints, known or new problems, sick leave, and other medical certificates (Fig. 2). Respondents were most likely to imagine receiving help from MPAs exclusively for vaccination/vaccination advice (40.2%) and prescription (33.4%) (Fig. 2).

### General acceptance of task delegation, regardless of the current problem/request

**Fig. 3:**
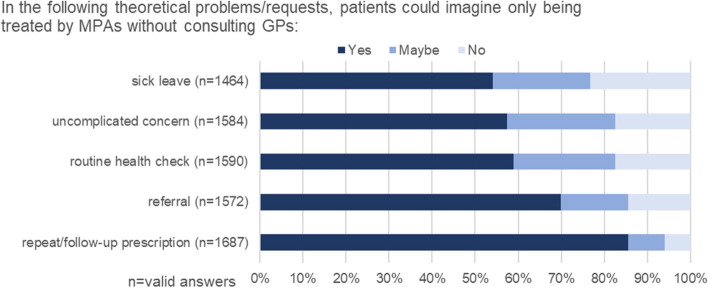
Theoretical problems/requests and patients’ acceptance of task delegation

The willingness to be treated exclusively by MPAs was also surveyed in relation to five theoretical reasons for care. For all five scenarios, over 54% of respondents expressed their agreement with care being provided by MPAs (Fig. 3). The highest level of agreement was obtained for the scenarios “repeat prescription/follow-up prescription” (85.6%) and “referral” (69.9%) (Fig. 3). The most frequent free-text responses on other reasons could be categorised as “discussion of medical findings”, “vaccination/vaccination consultation”, “examinations” and “health complaints”.

### Multivariable analyses

**Fig. 4:**
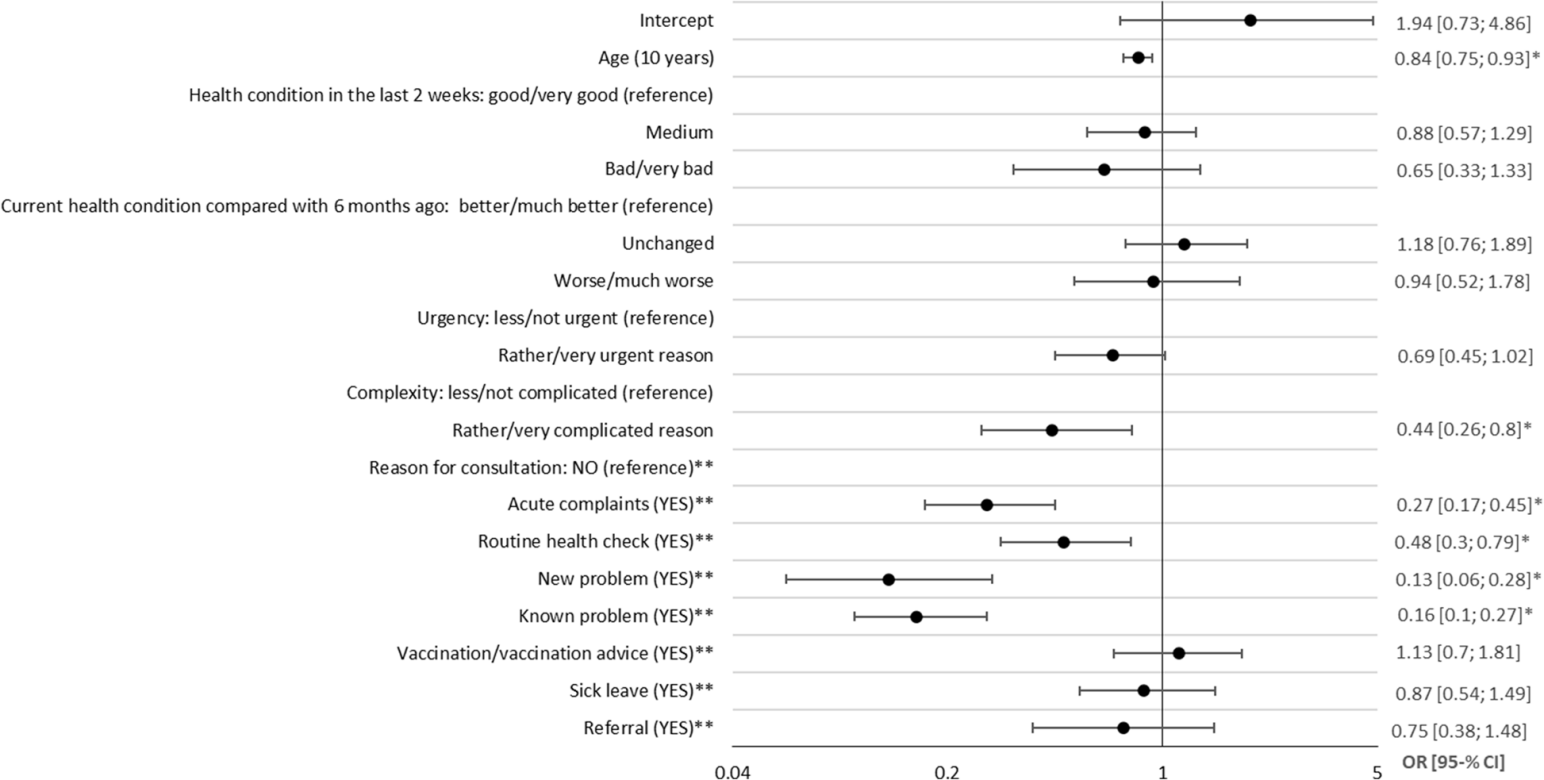
Forest plot mixed binary logistic model (random intercept model) for patients’ acceptance of task delegation. Analysis is based on information from 1861 patients from 61 practices. The dependent variable was patients’ acceptance of delegation processes: For my present concern…do I absolutely have to speak to the GP (coded as 0), …could the MPAs help me without talking to the GP (coded as 1). * *p*-value < 0.05; ** The reference are patients who have not ticked this reason for consultation

Multivariable analysis was used to adjust for possible confounding. For the present model, the ICC was 12.7%, which corresponded to substantial differences in task delegation acceptance responses between practices. The model showed an effect of age (10-year-OR 0.84, 95%-CI [0.75, 0.93]) and subjectively perceived complexity (OR = 0.44, 95%-CI [0.26, 0.8]) of the current concern on delegation acceptance (Fig. 4). Younger age and reporting a less complicated problem/request were associated with a higher willingness to seek care exclusively from MPAs instead of GPs. Furthermore, patients were more likely to seek care from GPs with the following problems/requests: “acute complaints” (OR = 0.27, 95%-CI [0.17, 0.45]), “routine health check” (OR = 0.48, 95%-CI [0.30, 0.79]), “new problem” (OR = 0.13, 95%-CI [0.06, 0.28]) and “known problem” (OR = 0.16, 95%-CI [0.10, 0.27]) (Fig. 4).

## Discussion

Our study explored the willingness of patients to be treated exclusively by MPAs instead of GPs in German general practices. We distinguished between the current reason for treatment and theoretical reasons for care. In total 1861 questionnaires from 61 practices were analysed.

When the patients were asked about their willingness to be treated exclusively by MPAs based on the given theoretical problems/requests, the majority agreed with this option. The high rates of agreement for theoretical care are comparable with the main result of Mergenthal et al. [31], where 65.9% of respondents could imagine MPAs taking on “other tasks”. Here only chronically ill patients and patients older than 65 years were included, and “further tasks” were not specified. The results of the German KBV telephone survey from 2017 also point in the same direction [32]. However, in this study only statements on so-called “minor illnesses” (67.2% agreement) and “chronic illnesses” (51.8% agreement) were asked. The Cochrane review by Karimi-Shahanjarini et al. [20] showed that patients prefer to be treated by physicians instead of MPAs for medical concerns (e.g., diagnosis of a serious illness, referrals). This result is not in line with the high acceptance of task delegation towards MPAs concerning the theoretical occasions given in our study. In contrast, our results are consistent in terms of the high delegation acceptance rates for repeat prescriptions/follow-up prescriptions and routine health checks presented by the Karimi-Shahanjarini et al. [20]. It is possible that patients overestimate their consent to delegation processes when asked in a more abstract and theoretical way. The lack of direct reference to the personal occasion of care and the subjective feeling or burden of discomfort associated with it could lead to these situational factors being disregarded when answering the question. Salisburg et al. [39] confirmed differences in response behaviour between general questions and questions specifically related to patients’ experiences. In the context of patient surveys, it is therefore advisable to ask individual questions about personal experiences instead of standardised questions (and answer categories) [40].

When asked about current concerns, patients’ approval of delegation seemed to be more modest (30% on average). This could also be due to multiple answers being possible here. In the case of multiple concerns, it remains unclear which reason was the most important and had the greatest influence on task delegation acceptance or rejection.

The mixed binary logistic regression analysis with inclusion of practice cluster effects showed that older patients and those with a problem/request subjectively perceived as complicated preferred to be treated by GPs instead of MPAs. Other patient characteristics that have been shown to be associated with the decision in other studies, such as the presence of a chronic condition, educational background [22, 32], or gender [39], were not confirmed by our study. Patients also preferred to be treated by GPs instead of MPAs for acute complaints, routine health checks, new or already known problems.

12.7% of the differences in response behaviour about delegation acceptance can be attributed to practice differences. A reason could be that in the practices, medical tasks are already delegated to MPAs to varying degrees. Thus, some patients may take it for granted that they will also receive follow-up prescriptions, referrals, or vaccinations from MPAs. For other patients this would be unusual, so they react with a reserved acceptance of delegation. In a survey of German GPs, Urban et al. found that 60% of the participating physicians are already supported by non-physician practice staff in issuing prescriptions for long-term medication or in delivering routine preventive services [11]. The fact that the practices are staffed differently could also be causal for the cluster effects at practice level. Accordingly, delegation could depend on the skill requirements and number of employed MPAs – for example, higher skill levels of MPAs could be associated with greater delegation of activities by GPs. However, Salisbury et al. found that the majority of practice-level factors (e.g., practice size, number of MPAs) predominantly did not significantly influence patient responses [39].

The strength of this study is the specific question of patients’ acceptance of task delegation in relation to theoretical and current health care occasions. In summary, the acceptance or rejection of task delegation depends on the reason for treatment [20] and its subjective evaluation by patients. These data, in addition to GPs and MPAs assessments [11, 12, 26, 34–38], bring an important patient perspective to the current debate on the expansion of delegation agreements and the need for new care models. The “German Medical Assembly”, the annual meeting of the “German Medical Association” (the central organisation in the system of medical self-administration in Germany) already has expressed its opinion several times against the expansion of delegable medical tasks and substitution processes [41, 42]. Although the occasions surveyed here are for the most part beyond the German delegation agreement and rather represent a substitution of GP’s service [8], they are not met with complete rejection by patients. The demographic development of declining numbers of physicians illustrates the need for new care concepts in primary care. For the first time, the results presented allow specific statements on the acceptance of task delegation by patients in relation to their actual and theoretical reasons for treatment to be made.

## Limitations

A selection bias among the participating GP practice teams cannot be completely ruled out – possibly practices interested in research are more open to new care models. The questionnaires were distributed to the patients by the practices themselves during consultation hours. Although the participating practices were specifically instructed not to distribute the questionnaire to certain patients, this cannot be ruled out with certainty.

Finally, 25.9% (*n* = 61) of the interested GPs (*n* = 235) took part. Referring to all GPs who had the opportunity to participate and who were contacted (*n* = 5516), only 1.1% took part. The achieved response rate was well below the broad range reported so far for primary care [43–45]. In this study, the response rate of GPs does not refer to the completion of questionnaires, but to general study participation as a recruitment site for patients. For patients’ response rate, it should be taken into account that each practice could only participate with a maximum of 50 questionnaires, and it remains open whether all questionnaires were handed out in the practices. This resulted in a response rate that varied greatly from practice to practice.

Despite anonymous data collection and piloting of the questionnaire, response bias cannot be completely ruled out. It is possible that patients answered according to social desirability, i.e., they tended to answer more positively or more critically than they would in reality. Maybe patients wanted to express more acceptance towards either the GPs or MPAs.

Finally, the generalisability and transferability of the data to the primary care delivered by GPs in the German healthcare system – and thus the representativeness of the obtained sample – must be limited, since the study was conducted exclusively in three German federal states. At the same time, Berlin, Brandenburg, and Thuringia represent 10% of the total German population [46]. Thus, the diversity from metropolitan to rural regions, as well as the regional and transregional differences in the socio-economic status of the population, are represented [47, 48].

## Conclusion

The patient perspective seems not to conflict with an expansion of the delegation of medical activities to the point of substitution by MPAs. It rather affirms new solutions for optimised work distribution and relief in the practice teams.

Future implementation processes of new or extended care concepts could benefit from actively including patients’ perspectives on the individual care situations. An introductory question at the beginning of the practice visit as to who should be providing care (GP or MPA) – as already suggested by Egidi et al. [49] – could provide transparency in the care process and involve patients. Future interventional studies should investigate the impact of delegation on patient safety, quality of care, the physician–patient relationship and the satisfaction of all parties involved. Furthermore, it should be defined which “minor illnesses” are most likely to be delegated [49].

It has also been shown that questions about specific reasons for consultation are answered more meaningfully than theoretical questions. In future patient surveys, it is advisable to ask questions as specifically, close to everyday life, and situation-related as possible.

## Data Availability

The datasets used and/or analyzed during the current study are available from the corresponding author on reasonable request.

## Abbreviations

AGnES: General practitioner-supporting, community-based, e-health-assisted, systematic intervention in Germany
CI: Confidence interval
GPs: General practitioners
KBV: National Association of Statutory Health Insurance Physicians
MPAs: Medical practice assistants
NPs: Nurse practitioners
ORs: Odds ratios
PAs: Physician associates/assistants
SHI: Statutory health insurance
VERAH: Health care assistants in general practices in Germany
WHO: World Health Organization
